# Predicting the Current and Future Habitat Distribution for an Important Fruit Pest, *Grapholita dimorpha* Komai (Lepidoptera: Tortricidae), Using an Optimized MaxEnt Model

**DOI:** 10.3390/insects16060623

**Published:** 2025-06-12

**Authors:** Li Huang, Shichao Zuo, Yiqi Huo, Lizong Hu, Zhengbing Wang, Jiahui Zhang, Jin Liu, Weili Ding, Keshi Ma, Mingsheng Yang

**Affiliations:** 1 College of Life Science and Agronomy, Zhoukou Normal University, Zhoukou 466001, China; yananhl@163.com (L.H.); 13394849655@163.com (S.Z.); huoyq22@126.com (Y.H.); hulizong@126.com (L.H.); wangzb@zknu.edu.cn (Z.W.); zhangjiahui0806@163.com (J.Z.); 20242008@zknu.edu.cn (J.L.); 2 Field Observation and Research Station of Green Agriculture in Dancheng County, Zhoukou 466001, China; 3 Finance Office, Zhoukou Normal University, Zhoukou 466001, China; dingyi77@163.com

**Keywords:** *Grapholita dimorpha*, Tortricidae, maximum entropy, climate change, species distribution model

## Abstract

The *Grapholita dimorpha* is one of the significant borer pests that primarily damage plum, pear, and apple trees, often resulting in substantial economic losses in fruit production. However, the potential distribution range of this economically important pest remains poorly understood. In this study, we simulated an optimized maximum entropy (MaxEnt) model to predict the spatiotemporal distribution pattern of *G. dimorpha* and identified its underlying driving factors. The results indicate that suitable habitats, under current bioclimatic conditions, are mainly distributed in eastern China, northeastern China, Korea, and Japan, covering a total of 273.5 × 10^4^ km^2^. The highly suitable habitats are primarily located in Korea and parts of central Japan, with a total area of 19.8 × 10^4^ km^2^. In future projections, the suitable area is expected to increase by 17.74% to 62.10%, and the suitable habitats are predicted to shift northward overall. In particular, there are more highly suitable habitats for *G. dimorpha* in China and Japan compared to their predominance in Korea under current climatic conditions. The bio9 and bio18 contribute 51.9% and 20.7% to the modeling, respectively, indicating that the distribution of *G. dimorpha* may be shaped mainly by the mean temperature of the driest quarter and precipitation of the warmest quarter. In summary, the distribution range predicted, particularly for regions with highly suitable habitats, poses a high risk of *G. dimorpha* outbreaks, emphasizing the priority of pest monitoring and management. Furthermore, the key bioclimatic variables identified could also provide crucial reference for pest monitoring.

## Simple Summary

The *Grapholita dimorpha* Komai (Lepidoptera: Tortricidae) is an important agricultural pest that seriously affects fruit production in Asia. In this study, for the first time, we predicted the potential habitat distribution of this pest using an optimized maximum entropy (MaxEnt) model. Under current bioclimatic conditions, the suitable habitats for *G. dimorpha* are primarily distributed in eastern China, northeastern China, Korea, and Japan, with the highly suitable habitats in Korea and parts of central Japan. In the future, the suitable habitats are predicted to shift northward overall, and in China and Japan, there are more highly suitable habitats. The bioclimatic factors bio9 (mean temperature of the driest quarter) and bio18 (precipitation of the warmest quarter) are the key variables affecting the potential distribution of *G. dimorpha*.

## 1. Introduction

*Grapholita dimorpha* belongs to the Tortricidae family within the order Lepidoptera and is an economically significant pest to various fruits, primarily plum, pear, and apple [1,2,3,4]. The female *G. dimorpha* typically lays one or two eggs on the fruit surface. After hatching, the larva creates a pinhole in the fruit skin and bores into the fruit [5]. The larva remains within the fruit until harvest. The presence of larvae within fruits is usually difficult to detect because the pinhole is too small, leading to consumer complaints in fruit markets [4]. In pest management, since *G. dimorpha* hides within the fruit from the first instar until the pupal stage, it is challenging to control with insecticidal sprays, and the application of sex pheromone has been investigated and utilized [4,6].

*Grapholita dimorpha* was first described in 1979 by Treitschke, with the type specimens collected from Japan [7]. Subsequently, it was also found in China [8], Korea [9], and Russia [10]. As a result, *G. dimorpha* is considered to have a distribution range in northeast Asia, including China, Korea, Japan, and Russia [3,11]. However, this range is merely inferred from point distribution records of *G. dimorpha* from the aforementioned taxonomic literature, based on sampling records of examined specimens or field surveys. These records do not indicate the spatial distribution pattern or distribution dynamics over time [12,13]. Such information is typically required to guide targeted pest management and pest monitoring [13,14]. In terms of taxonomy, *G. dimorpha* is a sibling species of the congeneric pest *G. molesta*, and they are extremely similar in morphology, largely sharing the same host plants, and can even be attracted to the same commercial sex pheromone lures in fruit production [2,6,7]. *G. molesta*, also known as the oriental fruit moth, is notorious for its global invasion from its native region (East Asia) to other continents, and it has become a cosmopolitan pest of stone and pome fruits [15,16]. Although *G. dimorpha* has not been regarded as an important invasive pest, its close similarity to *G. molesta* suggests that *G. dimorpha* possesses a significant potential for becoming an invasive species. Therefore, an improved understanding of the spatiotemporal distribution of *G. dimorpha* is necessary for pest management and the evaluation of the invasiveness potential of *G. dimorpha*.

To uncover the spatiotemporal distribution patterns of a species, one of the most popular methods is the use of species distribution models (SDMs). These models relate the occurrence records of a species to a set of environmental variables to predict its distribution across the study area [12,17,18]. A variety of modeling algorithms have been developed, such as the maximum entropy model (MaxEnt) [19], support vector machine (SVM) [20], generalized linear model (GLM) [21], and random forest (RF) [22]. Among the SDM procedures, the MaxEnt (maximum entropy) method utilizes a machine-learning algorithm based on the principle of maximum entropy to predict the habitat suitability of a specified species [19,23]. This modeling approach is distinguished by its superior prediction validity with a small sample size and straightforward interpretation of prediction results, and it has been extensively applied to various species, including insect pests [24,25,26,27]. In MaxEnt modeling, feature combinations (FCs) and regularization multipliers (RMs) are critical hyperparameters. By optimizing these FC and RM parameters, the model can enhance predictive accuracy and generalization ability, extract crucial information from environmental variables, and decrease sensitivity to noise or non-representative data [28]. In recent years, this method has been increasingly used in SDM studies on insect pests (e.g., [13,29,30]). For the economically significant *G. dimorpha*, previous research has concentrated on taxonomy and molecular identification [1,31,32,33], genomics and population genetics [34,35,36], and pheromone studies [4,6]. To date, however, no investigation has been conducted to predict the potential distribution of *G. dimorpha* habitats using the SDM method, and the key factors influencing the distribution pattern of this pest remain to be elucidated.

Given its significant effects on fruit production, this study employed the optimized MaxEnt algorithm [19] to predict the spatiotemporal distribution patterns of *G. dimorpha*. The objectives of the study were as follows: (i) to predict the current distribution patterns of suitable habitats for *G. dimorpha*; (ii) to understand the potential spatiotemporal distribution dynamics of this pest under various climate change scenarios; and (iii) to identify the major driving factors underlying the potential distribution of this pest.

## 2. Materials and Methods

### 2.1. Occurrence Records

**Figure 1:**
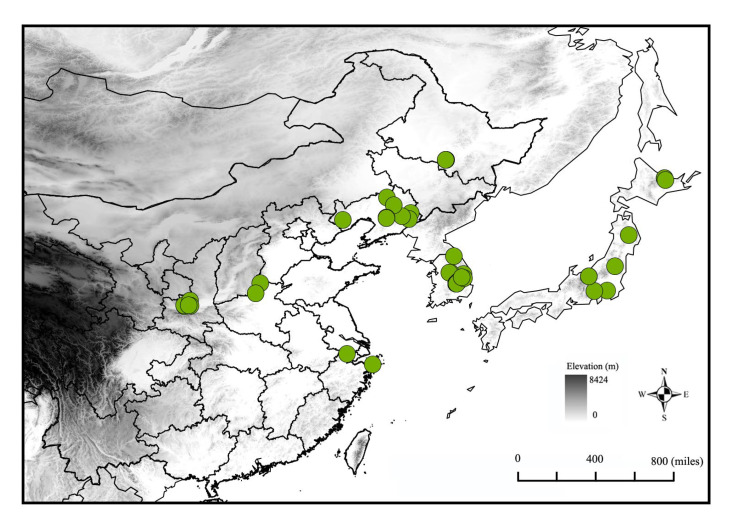
The occurrence records of *Grapholita dimorpha* used in the modeling.

We compiled occurrence records of *G. dimorpha* from three sources: the Global Biodiversity Information Facility database (GBIF, https://www.gbif.org/species/1736497, accessed on 10 July 2023), the Barcode of Life Data system (BOLD, http://v4.boldsystems.org/, accessed on 10 July 2023), and published literature from the China National Knowledge Infrastructure (CNKI, https://www.cnki.net, accessed on 10 July 2023). In the literature, distribution information lacking coordinates but indicating specific localities, such as town names, was considered and converted into coordinate data using the website https://api.map.baidu.com/lbsapi/getpoint/ (accessed on 3 October 2023). Initially, we compiled a total of 52 occurrence records, with 22 from GBIF, 2 from BOLD, and 28 from CNKI. Subsequently, we employed the clean_coordinates function from the R package “Coordinate Cleaner” [37] to eliminate redundant distribution data or erroneous coordinates, such as those situated in oceans, national capitals, and biodiversity institutions. Furthermore, the occurrence records were refined using the R package “spThin” [38] to ensure that only one occurrence record was present in each grid cell, thereby reducing spatial bias that could lead to model overfitting due to sampling bias [39,40]. Consequently, 33 occurrence records (Figure 1, Table S1) were utilized for modeling.

### 2.2. Bioclimatic Variables

We downloaded the 19 typical climate-associated environmental layers from WorldClim 2.1 (https://www.worldclim.org/, accessed on 9 June 2025) [41]. Given the possibility of collinearity among variables, which may lead to spatial autocorrelation [42], we conducted variable screening. During this process, we performed a Pearson’s correlation analysis using the R package “corrplot” [43]. If the absolute value of the correlation coefficient between two variables exceeded 0.8 [44], one of them was randomly removed in the R procedure.

We used the near-current bioclimatic layers representing the period from 1970 to 2000 to project the current potential distributions of suitable habitats of *G. dimorpha*. For future projections, we employed bioclimatic layers corresponding to the periods of 2021–2040 (2023s), 2041–2060 (2050s), and 2061–2080 (2070s), under both low (ssp126) and high (ssp585) greenhouse-gas-emission scenarios from the Coupled Model Intercomparison Project 6 (CMIP6) version. To enhance projection accuracy, for each time period, we chose three globally recognized circulation models (GCMs) (BCC-CSM2-MR, IPSL-CM6A-LR, and MRI-ESM2-0), which represent varying climate sensitivities to future climate change projections [13,14,45,46]. All environmental layers were developed at a spatial resolution of 2.5 arc-minutes.

### 2.3. Model Selection and Setting

We selected the MaxEnt procedure, which employs a machine-learning algorithm, to conduct our projection analyses [19,23]. In this process, the combination of feature classes (FCs) and regularization multipliers (RMs) was optimized to avoid overfitting and improve transferability [47]. We performed the optimization using the R packages “ENMeval” [47,48] and “dismo” [49]. Six FCs (L, H, LQ, LQH, LQHP, and LQPHT) and eight RMs (ranging from 0.5 to 4.0 in intervals of 0.5) were set to calculate the standardized Akaike information criterion coefficient (AICc). We selected the combination of FC and RM with the lowest delta AICc score for MaxEnt modeling. For other settings, we randomly set 75% of the occurrence records as training data and the remaining 25% for model validation. Ten replicates were conducted for each analysis, with maximum iterations set to 5000 and background points set to 10,000. We chose the “create response curves” and “do jackknife to measure variable importance” options to evaluate the impacts of variable changes on the presence probability of *G. dimorpha* and variable contribution, respectively.

### 2.4. Model Evaluation and Analyses

We utilized the area under the curve (AUC) within the receiver operating characteristic (ROC) curve to assess model performance. The AUC values were generated using Maxent version 3.4.1 software, ranging from 0 to 1. According to previous studies [50,51], an AUC of 0.7–0.8 indicates the model performance is acceptable, 0.8–0.9 that it is great, and an AUC greater than 0.9 is considered remarkable. When the AUC is below 0.5, the model’s performance is deemed no better than random. Additionally, we calculated the true skill statistic (TSS) values using the R package “ecospat” [52] to further evaluate model performance. The TSS values ranged from −1 to +1, with values approaching 1 indicating perfect predictive performance, while values of zero or less suggest that the model’s performance is no better than random [53,54].

The habitat suitability in the prediction maps generated by the MaxEnt procedure was continuous. We used the maximum training sensitivity plus specificity (MTSPS) logistic threshold value to define the suitable and unsuitable habitats for *G. dimorpha*, following the methodology of previous studies [13,14,55,56]. As such, the habitat suitability on the prediction maps was divided into four levels according to the threshold value. Regions with a probability less than the threshold were considered unsuitable, while suitable habitats with a distribution probability greater than the threshold were further classified into three levels: lowly, moderately, and highly suitable. We visualized the index of percent contribution and the jackknife value from the MaxEnt procedure to depict the impact or importance of environmental variables on the projections. Furthermore, we utilized ArcGIS 10.4 (Esri, Redlands, CA, USA) to calculate the areas of suitable regions predicted under current and future conditions.

## 3. Results

### 3.1. Variable Selection, Model Parameters, and Model Performance

**Figure 2:**
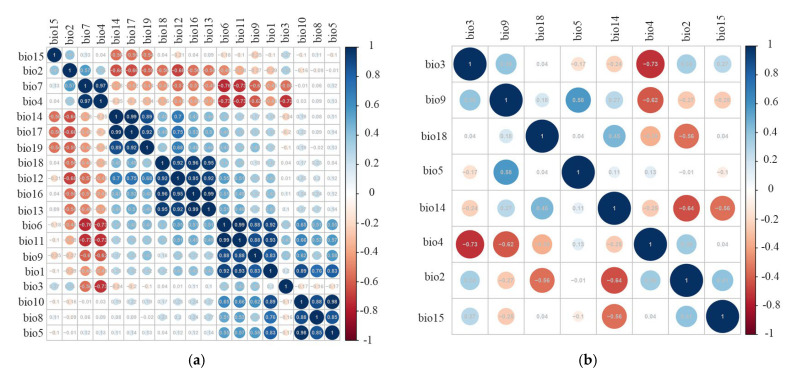
Pearson’s correlation analyses among the variables considered in this study. (**a**) Nineteen bioclimatic variables; (**b**) the eight bioclimatic variables.

**Figure 3:**
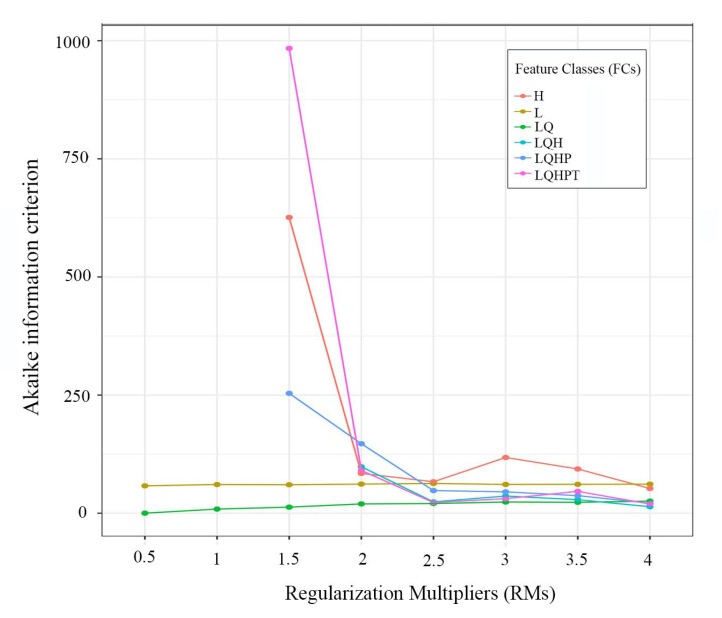
The model parameters used in the modeling. L, linear; Q, quadratic; H, hinge; P, product; T, threshold.

**Figure 4:**
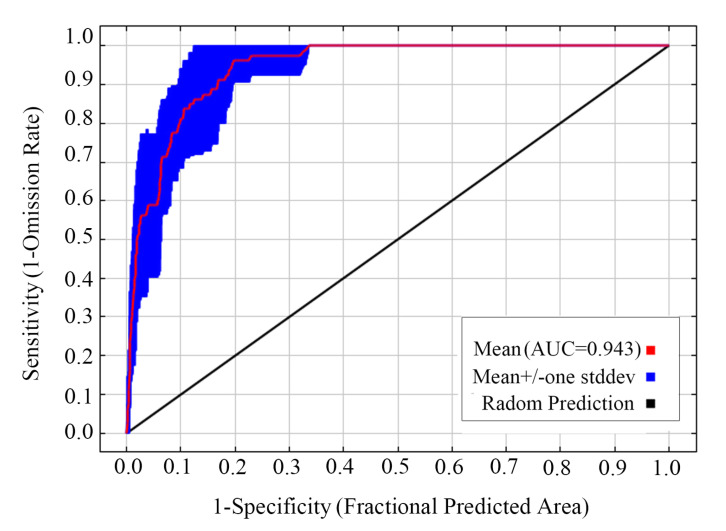
Receiver operating characteristic (ROC) curves and values of the area under the curves (AUC) of the modeling.

An analysis of Pearson’s correlation coefficient (Figure 2) selected 8 of the 19 bioclimatic variables for inclusion in modeling procedures. These variables included bio2 (mean diurnal range), bio3 (isothermality), bio4 (temperature seasonality), bio5 (maximum temperature of the warmest month), bio9 (mean temperature of the driest quarter), bio14 (precipitation of the driest month), bio15 (precipitation seasonality), and bio18 (precipitation of the warmest quarter) (Table S2). When the AICc was 0, the RM = 0.5 and FC = LQ represented the optimal parameter combination for MaxEnt modeling (Figure 3). The average test AUC value for the ten replicate runs was 0.943 (Figure 4), with a standard deviation of 0.024. The TSS value was 0.56. Both AUC and TSS indicated that the model performance was remarkable, and the projection results were reliable.

### 3.2. Variables’ Importance in the Modeling

**Figure 5:**
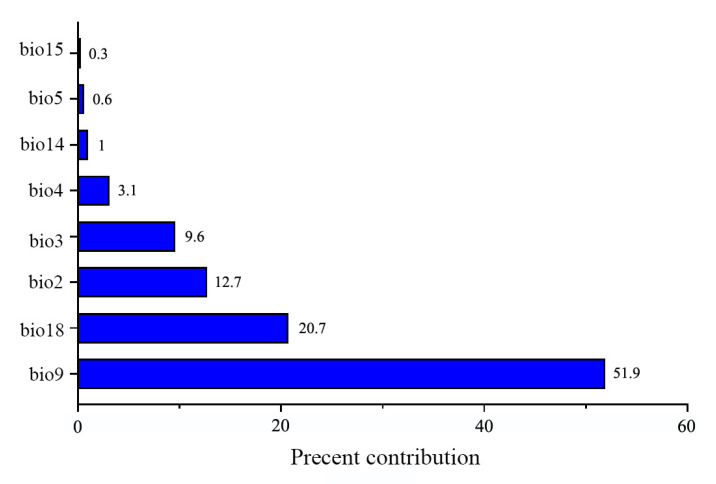
Percent contribution of the environmental variable used in the modeling.

**Figure 6:**
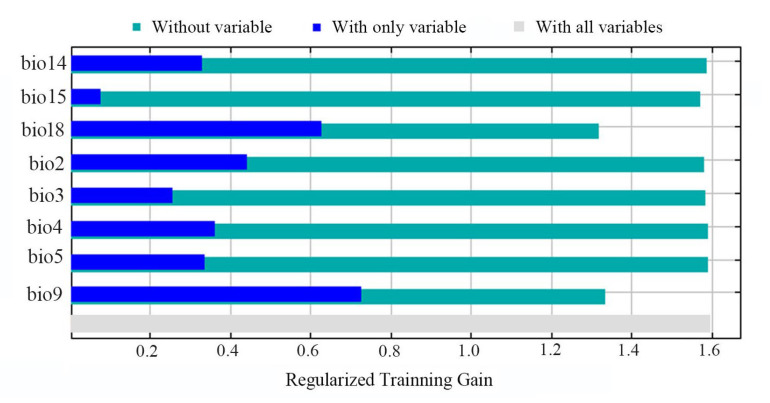
Results of the jackknife test. Variables with a longer blue bar or shorter green bar are considered to have greater relative importance in the modeling.

The contributions in terms of percentages for each variable to MaxEnt modeling are shown in Figure 5. Bio9 was the highest predictor (51.9%) contributing to the projection, followed by bio18 (20.7%), bio2 (12.7%), bio3 (9.6%), bio4 (3.1%), bio14 (1%), and bio5 (0.6%). Bio15 had the lowest contribution (0.3%). In general, bio9 represented the predominant variable and showed a significantly high contribution to the modeling. The pattern of variable importance revealed by jackknife analysis was generally identical to the result of percent contribution, especially on the definition of the top importance variables such as bio9, bio18, and bio2 (Figure 6). When the sole variable was considered, bio9, bio18, bio2, and bio4 were the top four rankings. In contrast, when bio9 and bio18 were excluded, the total contribution of other variables was much lower than that of all variables used, confirming the higher contribution of the two variables to the modeling.

### 3.3. Response Curves of the Top Four Contributing Variables Concerning the Presence Probability of Grapholita Dimorpha

**Figure 7:**
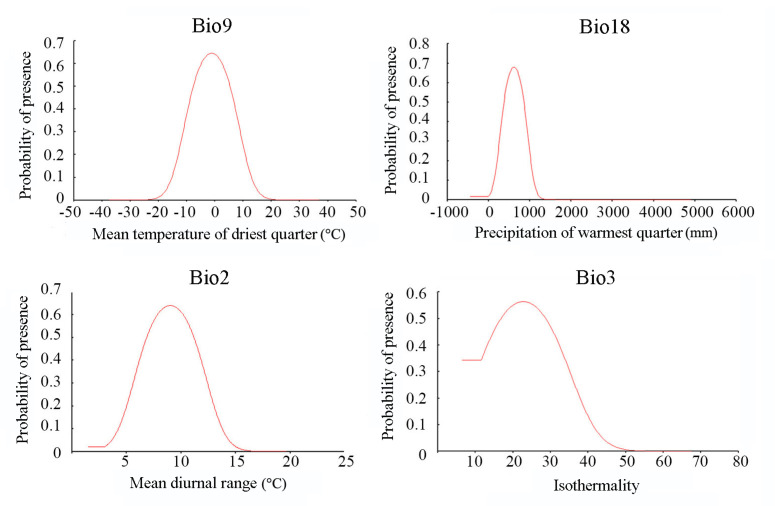
Response curves of the top four bioclimatic variables contributed to the modeling.

To understand the presence probability of *G. dimorpha* as a variable change, the response curves of the top four variables contributing to the model are illustrated in Figure 7. In general, the curves of all four predictors exhibited a unimodal distribution. For bio9 (mean temperature of the driest quarter), the presence probability increased with temperature from −20 °C, reaching the highest of 0.65 when the temperature was approximately −0.76 °C. Thereafter, the presence probability declined to 0 at about 20 °C. When the presence probability remained above 0.28, corresponding to the MTSPS threshold value, bio18 (precipitation of the warmest quarter) ranged between 255 mm and 985 mm, and reached its highest value of 0.68 at 678 mm. Starting from about 3 °C of bio2 (mean diurnal range), the presence probability of *G. dimorpha* began to increase, reaching its highest value of 0.64 at 9 °C, and then declined as the temperature of the mean diurnal range increased to about 18 °C. For bio3 (isothermality), the initial presence probability of *G. dimorpha* was 0.34, with isothermality ranging from 6.5 to 11.5, increased to the highest of 0.56 at 22.76, and then slowly declined with further increases in isothermality.

### 3.4. The Potential Distributions of Grapholita Dimorpha Habitats

**Figure 8:**
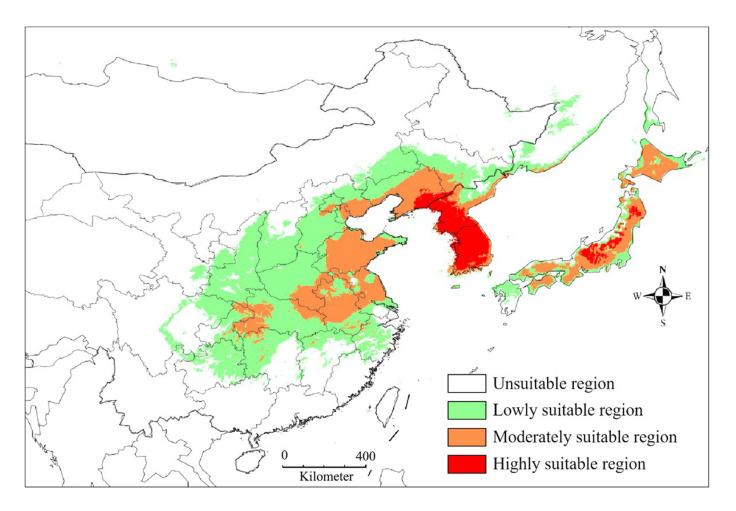
Predicted habitat suitability for *Grapholita dimorpha* under current bioclimatic conditions. 0–0.282: unsuitable, 0.282–0.521: lowly suitable, 0.521–0.761: moderately suitable, 0.761–1: highly suitable.

Suitable habitats, inferred under current bioclimatic conditions, are primarily distributed in eastern China, northeast China, Korea, and Japan, covering a total area of 273.5 × 10^4^ km^2^ (Figure 8, Table 1). The highly suitable regions are mainly distributed in Korea and parts of central Japan, with a total area of 19.8 × 10^4^ km^2^. Moderately suitable regions are primarily distributed in four Chinese provinces (Liaoning, Shandong, Anhui, and Jiangsu) and most of Japan, covering a total of 80.9 × 10^4^ km^2^. Consequently, most of the lowly suitable regions are defined in China, with a total area of 172.8 × 10^4^ km^2^.

**Figure 9:**
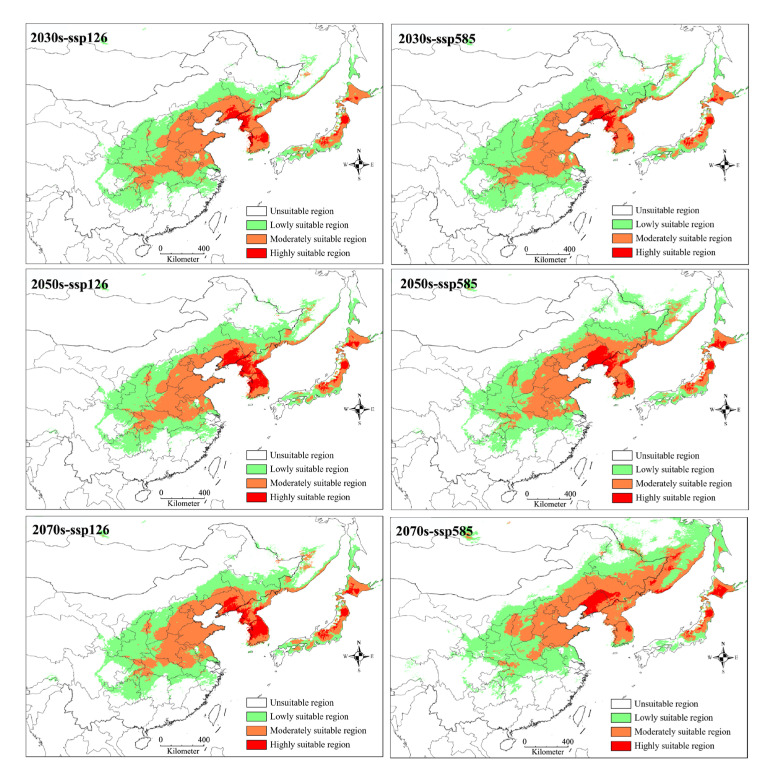
Predicted habitat suitability of *Grapholita dimorpha* under future climate change scenarios. 0–0.282: unsuitable, 0.282–0.521: lowly suitable, 0.521–0.761: moderately suitable, 0.761–1: highly suitable.

**Figure 10:**
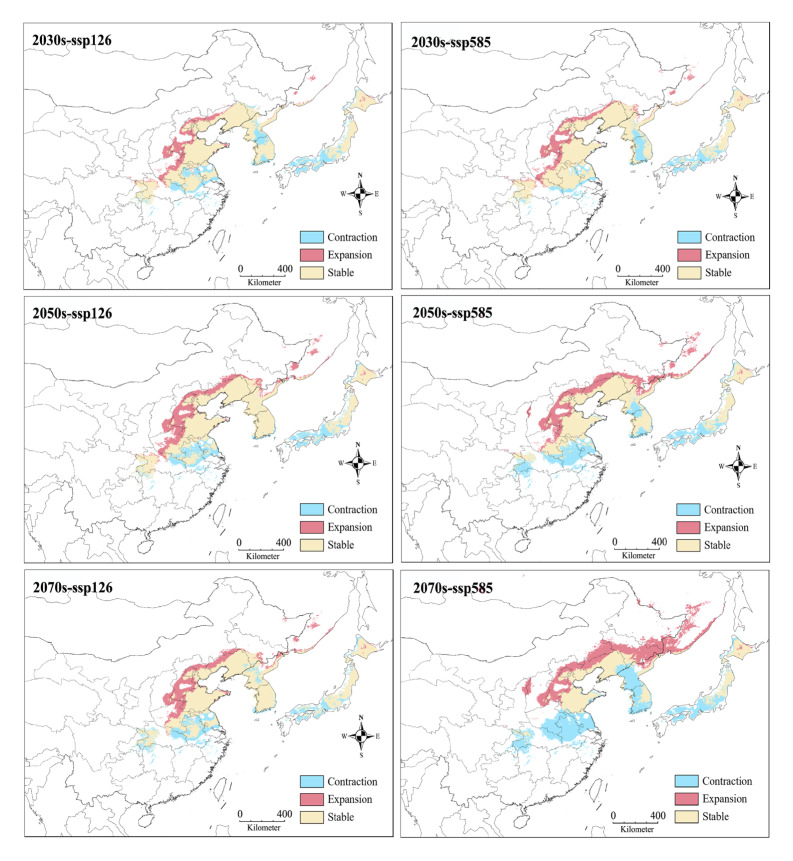
Projected change in suitable areas for *Grapholita dimorpha* under future scenarios relative to that under current conditions.

Under future climate change scenarios, all projections at different periods and greenhouse gas emissions consistently showed three characteristics (Figure 9 and Figure 10). First, the areas of suitable habitats were expanded compared with the current projection, and in particular, the moderately suitable area increased to the largest 165.5 × 10^4^ km^2^ at ssp585 of 2070s. Second, the distribution patterns of lowly, moderately, and highly suitable habitats had a significant change. For example, there were more habitats highly suitable for *G. dimorpha* in China and Japan, instead of predominantly in Korea under current bioclimatic conditions. Third, the distribution range of suitable habitats exhibited an overall movement towards the higher latitudes in China, whereas the southern edges of the suitable regions predicted under current bioclimatic conditions were generally not suitable for *G. dimorpha*.

## 4. Discussion

*Grapholita dimorpha*, akin to its close relatives *G. molesta* and *G. funebrana*, is one of the most significant borer pests affecting fruit production, particularly in Asia. In this study, we developed an optimized MaxEnt model to predict the spatiotemporal distribution pattern of *G. dimorpha* habitats for the first time. The values of the AUC and TSS indices indicate that the model performs well. Additionally, the general consistency between the distribution range defined by previous taxonomic records [3,11] and the current predictions suggests that the results are reliable.

Previous investigations indicate that the distribution range of *G. dimorpha* is primarily recorded in Japan, China, Korea, and Eastern Russia [7,8,9,10]. However, this range is inferred from scattered distribution points provided by the aforementioned publications, from which we cannot obtain detailed distribution information. For instance, it is unclear whether this pest is distributed in other localities beyond the scattered points, nor do we have insights into the spatial distribution possibilities [12]. Under current bioclimatic conditions, the MaxEnt model projected the potential habitat distribution of *G. dimorpha* primarily in China, Korea, and Japan. In China, *G. dimorpha* was first recorded in Heilongjiang Province of northeast China by Yan et al. [8], and then it was sampled in Shaanxi Province of northwest China, Henan Province of central China, Liaoning Province of northeast China, and Zhejiang Province of east China [32,33,57]. In contrast, our prediction results revealed a vast suitable area for *G. dimorpha*, mainly located in central China and part of northeast China. In some provinces, such as Shandong and Anhui in central China, where *G. dimorpha* has not been recorded, there is even a moderate habitat suitability. Furthermore, in South Liaoning Province, adjacent to Korea, there are highly suitable habitats, indicating a greater risk of *G. dimorpha* outbreaks. In Korea, *G. dimorpha* was first documented in 2009 by Choi et al. [9], and, subsequently, it has attracted significant attention due to its detrimental effects on fruits (e.g., [1,34,35]). Consequently, most of Korea is projected to have highly suitable habitats for *G. dimorpha*, suggesting a high potential for the occurrence or even outbreak of this pest. *Grapholita dimorpha* was first reported in Japan in 1978, with specimens sampled from Honshu [7]. Additionally, the distribution of *G. dimorpha* has been recorded in Iwate and Nagano, Japan [31]. Our prediction results indicate a broader distribution range, with most of Japan being moderately suitable for *G. dimorpha*, and parts of central Japan showing high suitability. Furthermore, *G. dimorpha* has been recorded in the southern Russian Far East [10], which aligns with our predictions, and in this region, suitable habitats have been projected. In summary, our results could more effectively aid in understanding the current spatial distribution patterns of *G. dimorpha*.

One of the key strategies for species, including invertebrate pests, to respond to future environmental changes, particularly climate warming, is range shifting (mostly poleward and upslope) to track favorable temperatures and/or meet moisture requirements [26,58,59]. Under future scenarios, most past SDM studies have revealed that pest species subjected to climate change would experience an expansion of suitable habitat. Examples include the hemipteran *Riptortus pedestris* [14], the dipteran *Aedes aegypti* [60], and the hymenopteran *Tamarixia radiata* [61]. However, an overall contraction of suitable habitat has also been reported for some pests, such as the lepidopteran *Spodoptera frugiperda* [62] and the hemipteran *Dalbulus maidis* [63]. For *G. dimorpha*, the total suitable areas for both ssp126 and ssp585 scenarios consistently increased compared to those under current climate conditions as the future period progressed. Generally, the areas deemed lowly and moderately suitable experienced significant expansion, while the highly suitable habitat remained stable. Furthermore, the distribution pattern of suitable habitats indicates a clear tendency towards northward expansion and southward contraction in future projections, aligning with a common result of poleward shifts for pests under future climate change [13,58,64]. Consequently, the expanded suitable habitats are predominantly located in China, and, notably, the area of highly suitable habitats for *G. dimorpha* is expected to increase in northeast China.

Among ecological factors, climate-related temperature and precipitation are often considered to have the most significant impact on the geographical range shifts of species [26,59,65]. This is likely because these factors are closely linked to the energy and water availability essential for species survival [14,66,67]. Consequently, in our present study, we aim to assess the effects of bioclimatic variables on the potential distribution of *G. dimorpha*. Of the eight bioclimatic variables chosen, bio9 (mean temperature of the driest quarter) was identified as the primary predictor influencing the projection, aligning with some SDM studies that suggest one or two variables are predominantly responsible for shaping the habitat distribution of a given species (e.g., [14,61,68]). To our knowledge, no field or laboratory research has been conducted on the effects of temperature on the survival of *G. dimorpha*. Our findings could serve as a guide for field monitoring. For example, areas where the mean temperature of the driest quarter is around −4 °C may have a higher risk of *G. dimorpha* outbreaks, as indicated by the response curve of bio9.

*Grapholita dimorpha*, an economically significant pest species, has garnered intense scrutiny due to its detrimental impact on the production of fruits such as plums, pears, and apples. Consequently, stringent surveillance and management strategies for this pest are urgently required. The present study predicts the potential distribution patterns of *G. dimorpha* under current and future climate change scenarios, offering a crucial reference for decision-making regarding this pest. For instance, regions particularly highly suitable for *G. dimorpha*, where fruit host plants are cultivated, merit close attention. Firstly, extensive field surveys or monitoring should be conducted promptly to confirm the presence of this species in these areas. This can be achieved through methods such as pheromone trapping [6] or DNA barcoding due to the high morphological similarity between *G. dimorpha* and its close relatives, such as *G. molesta* and *G. funebrana*, which may coexist sympatrically [32,33]. As for management strategies, beyond the use of chemical insecticides, pheromone traps, and an environmentally friendly management approach, have also been widely adopted [6,47]. In the future, similar to the management of *G. funebrana* [69,70], egg parasitoids like *Trichogramma* spp. should be developed as potential biological control agents for *G. dimorpha*. As such, the spatiotemporal distribution patterns of *G. dimorpha* predicted herein can significantly enhance the effectiveness of *Trichogramma* spp. in terms of release timing and locations, thereby more efficiently controlling *G. dimorpha*.

## 5. Conclusions

This study simulated an optimized MaxEnt model to predict the potential spatiotemporal distribution patterns of an economically important fruit pest, *G. dimorpha*, and identified the main bioclimatic factors shaping the distribution patterns. The results indicate that the current suitable habitats of *G. dimorpha* are primarily distributed across China, Korea, and Japan, with the highly suitable regions being distributed in most of Korea and parts of central Japan. Under future climate change scenarios, the distribution range of suitable habitats is projected to expand overall towards higher latitudes and contract southward. Overall, the areas of suitable habitats are expected to increase by 17.74% to 62.10% compared to the current suitable habitat area. Among the bioclimatic factors analyzed, bio9 (mean temperature of the driest quarter) contributes 51.9% to the projection, signifying its significant role in influencing the habitat distribution of *G. dimorpha*. The findings suggest that the risk of *G. dimorpha* outbreaks will persist in the future, and the spatiotemporal distribution patterns of suitable habitats and the shaping factors predicted in this study could offer crucial guidance for *G. dimorpha* management.

## Figures and Tables

**Table 1:** The change of suitable areas of *Grapholita dimorpha* under current conditions and future climate change scenarios (×10^4^ km^2^).

Decades/Climate Scenarios		Area (×10^4^ km^2^)			
		All Suitable Habitats	Lowly Suitable Habitats	Moderately Suitable Habitats	Highly Suitable Habitats
1970–2000		273.5	172.79	80.89	19.82
ssp126	2030s	322.03	183.73	124.88	13.42
ssp126	2050s	341.59	194.51	125.94	21.3
ssp126	2070s	350.1	202.37	127.82	19.91
ssp585	2030s	332.66	194.9	124.97	12.79
ssp585	2050s	376.9	224.63	132.8	19.48
ssp585	2070s	443.35	259.63	165.45	18.27

## Data Availability

The original contributions presented in this study are included in the article/Supplementary Material. Further inquiries can be directed to the corresponding authors.

## Supplementary Materials

The following are available online at https://www.mdpi.com/article/10.3390/insects16060623/s1; Table S1: The occurrence records of *Grapholita dimorpha* used in species distribution modeling; Table S2: The environmental variables considered in this study.
