# Supplementary material for: Predicting the Current and Future Habitat Distribution for an Important Fruit Pest, Grapholita dimorpha Komai (Lepidoptera: Tortricidae), Using an Optimized MaxEnt Model
Source: Insects. 2025 Jun 12;16(6):623. doi: 10.3390/insects16060623 (PMC12193446; doi:10.3390/insects16060623)
Supplement: Supplementary file 1 [file insects-16-00623-s001.zip › Table S2. The environmental variables considered in this study.pdf]

Table S2. The 19 bioclimatic variables considered in this study

**Variables**	**Descriptions**
Bio1	Annual mean temperature (°C)
**Bio2**	Mean diurnal range (mean of monthly (max temp-min temp)) (°C)
**Bio3**	Isothermality (bio2/bio7) (× 100)
**Bio4**	Temperature seasonality (standard deviation ×100)
**Bio5**	Max temperature of warmest month (°C)
Bio6	Min temperature of coldest month (°C)
Bio7	Annual temperature range (bio5–bio6) (°C)
Bio8	Mean temperature of wettest quarter (°C)
**Bio9**	Mean temperature of driest quarter (°C)
Bio10	Mean temperature of warmest quarter (°C)
Bio11	Mean temperature of coldest quarter (°C)
Bio12	Annual precipitation (mm)
Bio13	Precipitation of wettest month (mm)
**Bio14**	Precipitation of driest month (mm)
**Bio15**	Precipitation seasonality (coefficient of variation)
Bio16	Precipitation of wettest quarter (mm)
Bio17	Precipitation of driest quarter (mm)
**Bio18**	Precipitation of warmest quarter (mm)
Bio19	Precipitation of coldest quarter (mm)

Note: the variables in bold were finally used in the modeling.
